# Precision gene diagnosis and treatment of epilepsy: a new frontier in medical care

**DOI:** 10.1186/s42494-025-00241-3

**Published:** 2025-10-01

**Authors:** Jie Mu, Weijia Jiang, Ying Tang, Dong Zhou, Weiping Liao

**Affiliations:** 1 Neurology Department, West China Hospital of Sichuan University, 14# Third Section, Renmin Nan Road, Chengdu, 610041 China; 2 West China Medical Publishers, West China Hospital of Sichuan University, 14# Third Section, Renmin Nan Road, Chengdu, 610041 China; 3 Department of Neurology, Institute of Neuroscience, Key Laboratory of Neurogenetics and Channelopathies of Guangdong Province and the Ministry of Education of China, The Second Affiliated Hospital, Guangzhou Medical University, Guangzhou, 510260 China

Precision medicine serves as a beacon of hope, offering tailored treatments that target the unique molecular characteristics of individual patients. Among the numerous conditions that could benefit from this approach, epilepsy stands out as a particularly compelling target. In this special issue, we explore the promising potential of precision gene diagnosis or treatment for epilepsy, exploring its foundations, challenges, and future prospects.

Epilepsy is a prevalent neurological disorder that affects more than 9 million people in China. The etiology of epilepsy remains unknown in majority patients. Genetic studies indicate that genetic abnormalities play a pivotal role in the pathogenesis of epilepsy. Prior to the advent of precision genetic treatment, genetic testing had become accessible for individuals with epilepsy; however, interpreting the results of genetic tests remains a significant challenge.

The foundation of precision medicine lies in the ability to analyze and interpret a patient’s genetic information. Advances in genomics and bioinformatics have enabled the identification of specific genetic variants associated with epilepsy. These variants can influence the function or expression of genes involved in neuronal excitability, synaptic transmission, and other critical brain functions. By targeting these genetic alterations, precision gene treatment aims to restore normal brain activity and reduce or prevent seizures.

China Association Against Epilepsy (CAAE) launched the China Epilepsy Gene 1.0 (C-epg1.0) Project, aiming to make the genetic test helpful for diagnosis and management of epilepsy in clinical practice. As the director of C-epg1.0 Project, Professor Liao Weiping took the lead in this special issue drafting the topic of genes and epilepsy, and received many excellent manuscripts**.** The following representative manuscripts have opened a systematic introduction to the topic. Firstly, reviews introduce the latest developments in the topic. Secondly, two articles discuss the expansion of the clinical disease spectrum. Thirdly, potential novel causative genes are discovered. Finally, there are case reports on gene diagnosis and treatment.

The paper by Xiaoqian Wang et al. [1] titled “Genetic mechanisms in generalized epilepsies” presents a comprehensive review of the current understanding of the genetic factors contributing to genetic generalized epilepsies (GGEs). The study highlight the critical role of genetic factors in the etiology of GGEs. The authors discuss the diverse genetic mechanisms involved, including single gene variants, copy number variations, and common variants. These genetic alterations can lead to abnormalities in ion channels, neurotransmitters, and receptors, ultimately causing imbalance in neuronal excitability and synchronization, which are hallmarks of epileptogenesis. A significant focus of the paper is on identifying reliable genes for GGEs. Furthermore, the paper explores the therapeutic implications of these genetic discoveries, discussing potential precision treatments tailored to specific genetic profiles.

In another review article, Yuqing Shi et al. [2] provides a comprehensive overview of the relationship between disorders of organic acid metabolism and epilepsy. Organic acid metabolism disorders are a group of inborn errors of metabolism characterized by enzyme deficiencies that lead to the accumulation of toxic metabolites in the body. These disorders can cause a wide range of neurological symptoms, including epilepsy. The article discusses various organic acid metabolism disorders, such as methylmalonic acidemia, propionic acidemia, malonyl-CoA decarboxylase deficiency, and creatine deficiency syndrome, among others. The paper summarizes the genetic aspects of organic acid metabolism disorders associated with epilepsy, highlighting mutations in genes such as *MUT, PCCA, PCCB, MLYCD, SLC6A8, GAMT, AGAT, L2HGDH, D2HGDH, HTRA2, GCDH,* and *FH* and their pathogenic mechanisms, clinical features, diagnosis, and treatment options, with a particular focus on their association with epilepsy.

Another review article by Yajing Gan et al. [3] explores the intricate connection between vitamin metabolism disorders and epilepsy. Vitamins play crucial roles as coenzymes or components of enzymes involved in various biochemical processes within the body. Vitamin metabolism disorder-related epilepsy is a type of treatable metabolic epilepsy. Here is a summary of the diseases and their associated genes discussed in the article "Precision diagnosis and treatment of vitamin metabolism-related epilepsy”. Vitamin B6-responsive epilepsy relate to genes: *PLPBP, ALDH4A1, ALDH7A1, PNPO, TNSALP*; biotin-thiamine responsive basal ganglia disease (BTBGD) with *SLC19A3;* biotinidase deficiency with *BTD;* folate deficiency and gene: *FOLR1, SLC46A1, DHFR;* 3-phosphoglycerate dehydrogenase deficiency with gene *PHGDH*. This summary highlights the various genetic disorders linked to vitamin metabolism that can manifest with seizures, highlighting the importance of precise diagnosis and targeted treatment.

The paper “Characteristic spatial and frequency distribution of mutations in *SCN1A*” by Mengwen Zhang et al. [4] offers a detailed analysis of the characteristic spatial and frequency distributions of mutations in the *SCN1A* gene, which is widely recognized as one of the most commonly mutated genes associated with epilepsy. The *SCN1A* gene encodes the pore-forming α subunit of the neuronal voltage-gated sodium channel Nav1.1 and plays a critical role in brain function. By systematically reviewing mutations from various databases and literature, the authors identified distinct patterns in the distribution of *SCN1A* mutations. The study reveals that mutations tend to cluster at specific sites, influenced by CpG dinucleotides, exons, and functional domains of the Nav1.1 protein. The analysis provides valuable insights into the mutagenesis etiopathology of *SCN1A*-associated epilepsy, potentially guiding precision clinical management and therapeutic interventions.

Two papers refer the genes *SLC2A1* and *TSC1,* which are common causes of epilepsy. These studies expended the spectrum of the genes and are of clinical significance. This research paper by Dongming Zhang et al. [5] investigates the association between variants in the *SLC2A1* gene and late-onset epilepsy. The *SLC2A1* gene encodes glucose transporter type 1 (GLUT1), which is crucial for brain energy metabolism. Previous studies have linked *SLC2A1* variants to early-onset refractory seizures and glucose transporter type 1 deficiency syndrome (GLUT1DS). However, the relationship between *SLC2A1* and late-onset epilepsy remains unclear. Through trio-based whole-exome sequencing, the authors identified 14 heterozygous *SLC2A1* variants in 16 unrelated families with epilepsy. They found that while some variants were associated with early-onset developmental and epileptic encephalopathy (DEE), others were linked to late-onset epilepsy with normal development. The study also examined the temporal expression pattern of *SLC2A1*, which was found to be consistent with the seizure onset age, suggesting a genetic-dependent stage feature. The findings emphasize the importance of early genetic diagnosis in patients with *SLC2A1* variants.

The study by Nanxiang Shen et al. [6] titled “Variants of *TSC1* are associated with developmental and epileptic encephalopathy and focal epilepsy without tuberous sclerosis” investigates the potential association between *TSC1* variants and epilepsy, particularly in the absence of tuberous sclerosis complex (TSC). Through trio-based whole-exome sequencing in patients enrolled in the China Epilepsy Gene 1.0 Project, the authors identified *TSC1* variants in individuals with DEE and focal epilepsy. Specifically, two de novo *TSC1* truncating variants (c.1498 C > T/p.Arg500* and c.2356 C > T/p.Arg786*) were found in patients with DEE, while two heterozygous *TSC1* variants were detected in patients with focal epilepsy. These variants were absent from the gnomAD database and predicted to be pathogenic. Despite the absence of typical TSC symptoms and normal brain MRI findings, the identification of these *TSC1* variants in patients with epilepsy suggests that *TSC1* may play a role in common epilepsy, independent of TSC. This study provides new insights into the genetic etiology of epilepsy and the spectrum of *TSC1*-related disorders. This article reflects the important role of China Epilepsy Gene 1.0 Project in medical research. Two studies identified potential novel causative genes of epilepsy. The study by Wenwei Liu et al. [7] presents a novel finding regarding the association of *SPOUT1* variants with autosomal-recessive DEE. Through the identification of four unrelated Chinese patients carrying compound heterozygous *SPOUT1* variants, the authors demonstrate that *SPOUT1* is a candidate gene for DEE. All patients in the study exhibited early-onset seizures, microcephaly, and global developmental delay. Brain MRI revealed white matter hypomyelination and widened frontotemporal subarachnoid spaces in several patients. Functional analysis using a *spout1* knockout zebrafish model further supported the role of *SPOUT1* in epileptogenesis, revealing increased epileptiform signals and differential expression of genes related to axonal transport. These findings expand the genetic landscape of DEE and suggest potential mechanisms underlying the pathogenesis of this disorder.

The study by Qianru Wen et al. [8] identified *MDN1* as a potential susceptibility gene of epilepsy. Through trio-based whole-exome sequencing of patients from the China Epilepsy Gene 1.0 Project, compound heterozygous *MDN1* variants were identified in five unrelated patients with febrile seizures or secondary epilepsy. These variants were rare in controls and predicted to be damaging. The study suggests *MDN1* as a potential susceptibility gene for epilepsy, supported by the higher frequency of variants in cases compared to controls. The molecular subregional implication and hydrophobicity alterations of *MDN1* variants may play a role in epilepsy pathogenesis.

The case report by Tuo ji et al. [9] titled "De novo *ADGRV1* variant in a patient with ictal asystole provides novel clues for increased risk of SUDEP" presents a unique case of a 35-year-old man with drug-resistant epilepsy (DRE) who experienced ictal asystole, a rare and life-threatening cardiac arrhythmia during seizures. Ictal asystole, characterized by the absence of ventricular complexes for more than 4 s, is associated with an elevated risk of sudden unexpected death in epilepsy (SUDEP). Genetic testing uncovered a de novo variant in the Adhesion G Protein-Coupled Receptor V1 (*ADGRV1*) gene, which may provide clues to the patient's ictal asystole and increased SUDEP risk. This case highlights the importance of combined EEG-ECG monitoring in diagnosing ictal cardiac arrhythmias and underscores the potential role of genetic testing in identifying adult epilepsy patients at high risk for SUDEP.

This paper named “Effective treatment of *NR2F1*-related epilepsy with perampanel” by Xiao Li et al. [10] investigates the efficacy of perampanel in treating *NR2F1*-related epilepsy, a type of epilepsy associated with mutations in the *NR2F1* gene, which is also linked to Bosch-Boonstra-Schaaf optic atrophy syndrome (BBSOAS). The study presents six new Chinese cases of *NR2F1*-related epilepsy and summarizes 14 previously reported cases. The key finding is that perampanel, when used as an add-on treatment, effectively controlled seizures in all six new cases, demonstrating its potential as a novel and effective therapeutic option for *NR2F1*-related epilepsy. This research highlights the importance of genetic testing in identifying *NR2F1* mutations and the role of precision medicine in treating this complex condition.

One of the primary hurdles is the complexity of the human genome. Epilepsy is a highly heterogeneous condition with many different genetic causes. Identifying the specific genetic variants responsible for an individual’s epilepsy can be difficult and time-consuming. Despite these challenges, the future of precision genetic diagnosis and treatment for epilepsy remains promising.

## Data Availability

Not applicable.
